# Pharmacogenetic study of antipsychotic–induced lipid and BMI changes in Chinese schizophrenia patients: A Genome-Wide Association Study

**DOI:** 10.1038/s41398-025-03499-w

**Published:** 2025-08-19

**Authors:** Kenneth Chi-Yin Wong, Perry Bok-Man Leung, Benedict Ka-Wa Lee, Zoe Zi-Yu Zheng, Emily Man-Wah Tsang, Meng-Hui Liu, Kelly Wing-Kwan Lee, Shi-Tao Rao, Pak-Chung Sham, Simon Sai-Yu Lui, Hon-Cheong So

**Affiliations:** 1 School of Biomedical Sciences, The Chinese University of Hong Kong, Hong Kong SAR, China; 2 Department of Psychiatry, Li Ka Shing Faculty of Medicine, The University of Hong Kong, Hong Kong SAR, China; 3 Department of Psychosis Studies, Institute of Psychiatry, Psychology and Neuroscience, King’s College London, London, UK; 4 Department of Epidemiology, University of Florida, Gainesville, FL USA; 5 School of Medical Technology and Engineering, Fujian Medical University, Fuzhou, China; 6 Centre for PanorOmic Sciences, Li Ka Shing Faculty of Medicine, The University of Hong Kong, Hong Kong SAR, China; 7 State Key Laboratory of Brain and Cognitive Sciences, The University of Hong Kong, Hong Kong SAR, China; 8 KIZ-CUHK Joint Laboratory of Bioresources and Molecular Research of Common Diseases, Kunming Institute of Zoology and The Chinese University of Hong Kong, Hong Kong SAR, China; 9 Department of Psychiatry, The Chinese University of Hong Kong, Hong Kong SAR, China; 10 CUHK Shenzhen Research Institute, Shenzhen, China; 11 Margaret K. L. Cheung Research Centre for Management of Parkinsonism, The Chinese University of Hong Kong, Hong Kong SAR, China; 12 Brain and Mind Institute, The Chinese University of Hong Kong, Hong Kong SAR, China; 13 Hong Kong Branch of the Chinese Academy of Sciences Center for Excellence in Animal Evolution and Genetics, The Chinese University of Hong Kong, Hong Kong SAR, China

**Keywords:** Clinical genetics, Predictive markers, Schizophrenia

## Abstract

Second-generation antipsychotics (SGAs) are widely used to treat schizophrenia (SCZ), but they often induce metabolic side effects like dyslipidemia and obesity. We conducted genome-wide association studies (GWASs) to identify genetic variants associated with SGA-induced lipid and BMI changes in Chinese SCZ patients. A longitudinal cohort of Chinese SCZ receiving SGAs was followed for up to 18.7 years (mean = 5.7 years, SD = 3.3 years). We analysed the patients’ genotypes (*N* = 669), lipid profiles, and BMI using 19 316 prescription records and 3 917 to 7 596 metabolic measurements per outcome. Linear mixed models were employed to evaluate seven SGAs’ random effects on metabolic changes for each patient, followed by GWAS and gene set analyses with Bonferroni and FDR correction. Five SNPs achieved p-value < 5 × 10^−08^ before multiple testing correction: rs6532055 (*ABCG2*) linked to olanzapine-induced LDL changes, rs2644520 (near *SORCS1*) linked to aripiprazole-induced triglyceride changes, rs115843863 (near *UPP2*) linked to clozapine-induced HDL changes, rs2514895 (near *KIRREL3*) linked to paliperidone-induced LDL changes, and rs188405603 (*SLC2A9*) linked to quetiapine-induced triglyceride changes. These five SNPs passed FDR correction at 0.2 but not Bonferroni-corrected genome-wide significance threshold (p-value < 3.125 × 10^−10^) for 160 GWAS analyses. Gene-based analysis revealed six genome-wide significant genes after Bonferroni correction (p-value < 2.73 × 10^−6^): *ABCG2*, *APOA5*, *ZPR1*, *GCNT4*, *MAST2*, and *CRTAC1*. Four gene sets were significantly associated with SGA-induced metabolic side effects. In summary, this pharmacogenetic GWAS identified several genetic variants potentially associated with SGA-induced metabolic side effects, potentially informing personalized treatment strategies to minimize metabolic risk in SCZ patients. Given our limited sample size, further replications are required to confirm the findings.

## Introduction

Schizophrenia (SCZ) is a severe, chronic mental illness with high heritability and a lifetime prevalence of approximately 1%. The global burden of SCZ has been increasing, with the incidence increasing by 2% annually between 2000 and 2019 [1]. Cardiovascular disease (CVD) is the leading cause of mortality in SCZ patients [2], and psychosis itself is also a recognized risk factor for dyslipidemia and obesity [3]. Moreover, second-generation antipsychotics (SGAs), the mainstream treatment for SCZ, can adversely affect patients’ lipid profiles, other metabolic parameters, and body mass index (BMI) [4].

Interestingly, the propensity to develop these metabolic side effects varies considerably among individuals. Twin and sibling studies have demonstrated that such interindividual variability may be largely attributable to genetic differences [5]. However, the underlying genetic mechanisms remain poorly understood.

Pharmacogenetics (PGx) examines how genetic variations affect drug metabolism and response, potentially enabling personalised treatment plans. Over the past two decades, most PGx studies on SGA-induced metabolic side effects have employed candidate gene approaches, focusing primarily on dopamine and serotonin receptor-related genes [6, 7]. Additionally, variants in cytochrome P450 genes have been associated with antipsychotic serum concentrations [8]. Genome-wide association studies (GWASs) have largely overcome the limitations of candidate gene approaches, uncovering more variants and genes associated with antipsychotic response [9–11]. However, the majority of previous PGx studies have focused mainly on treatment response rather than the metabolic side effects of SGAs.

To date, only seven PGx GWASs have investigated SGA-induced metabolic side effects [12–18], with most focusing exclusively on weight gain and short-term outcomes. The most comprehensive study by Adkins et al. (2010) investigated various metabolic side effects across multiple antipsychotics over 18 months [12]. Nevertheless, this study had several limitations, including prior antipsychotic experience in most participants, concurrent use of other medications, potential bias in DNA collection, and lack of genotype imputation in GWAS analysis.

To address this knowledge gap, our PGx study investigated lipid and BMI changes induced by seven SGAs: olanzapine (OZP), clozapine (CZP), quetiapine (QUE), risperidone (RIS), aripiprazole (ARI), amisulpride (AMI) and paliperidone (PAL). We focused on BMI and four lipid measurements, including total cholesterol (TC), high-density cholesterol (HDL), low-density cholesterol (LDL) and triglycerides (TG), as outcomes. Our study utilized a longitudinal cohort with a longer follow-up of up to 5.7 years (median) and a greater mean number of metabolic measures per subject. The proportion of SGA-naïve patients was markedly greater at ~63%. Furthermore, the homogeneity of our Chinese cohort, recruited from Hong Kong, China, combined with imputed genotypes based on the ChinaMAP reference panel [19], enhanced the statistical power of GWAS to detect true signals.

This sophisticated approach combined with a long follow-up duration and comprehensive medication history and metabolic measures. We aimed to identify novel genetic variants associated with SGA-induced metabolic side effects. This approach may provide insights into the biological mechanisms underlying SGA-induced lipid and BMI changes, potentially contributing to more personalized and effective treatments for SCZ patients.

## Materials and methods

### Study population and data collection

We recruited SCZ patients from an early psychosis intervention clinic at Castle Peak Hospital Hong Kong between 2009 and 2021 [20]. The inclusion criteria were as follows: (1) aged ≥18 years, (2) Chinese ethnicity, (3) ICD-10 diagnosis of SCZ or schizoaffective disorder, (4) treatment with SGAs, and (5) at least one post-SGA measurement of fasting lipids and/or BMI. We excluded patients with preexisting metabolic disorders or those lost to psychiatric follow-up as of March 2021.

From 767 eligible patients, we extracted complete medication records, lipid profiles and BMI measurements from initial service contact to the study endpoint. Following local guidelines for monitoring SGA side effects, patients received baseline measures of fasting lipid profiles and BMI before SGA initiation, with annual follow-up measurements while on SGAs. Electronic health records documented the type and dosage of all psychotropic and concomitant medications, including antidepressants and lipid-lowering drugs.

### Genotyping and imputation

Blood samples from patients were genotyped using the Illumina Asian Screening Array-24 v1.0. Quality control was performed using PLINK 1.9p, removing genotypes and subjects based on missing data (missing rate > 10%), Hardy‒Weinberg equilibrium (*p* < 10^−6^), and relatedness (IBS distance > 0.25). No ethnic outliers were identified through multidimensional scaling, as shown in Supplementary Fig. S2.

The genome coordinates were lifted from GRCh37 to GRCh38 using CrossMap v0.6.4. Haplotype phasing and genotype imputation were conducted using Eagle2 and Minimac4, respectively [21, 22], with the ChinaMAP phase 1.v1 reference panel (59 010 860 sites from 10 155 Chinese individuals) [19]. This large, ancestrally matched reference panel improved imputation accuracy. The imputed SNPs were removed based on imputation quality (INFO score ≤ 0.3) and minor allele count (MAC ≤ 10). The final dataset comprised 6 992 805 high-quality imputed SNPs.

### Data preprocessing and variable selection

Full GWAS data were available for 669 SCZ patients with 19 316 prescription records within 3 months of metabolic measurement. We analysed TC, HDL, LDL, and TG (all in mmol/L), and BMI (kg/m^2^) as separate outcomes. To minimise bias in effect estimation, we excluded outliers that exceed six standard deviations from the group mean, as these values suggested potential measurement errors. We excluded 2 measurements each for TC, HDL, and BMI, and 11 measures for TG. No outliers were identified for LDL. The final analysis included 4 048 TC, 3 917 HDL, 4 035 LDL, 4 034 TG, and 7 596 BMI measurements. Prior to modelling, we applied natural log transformation to all measurements to better satisfy the normality assumptions of our linear mixed models **(**Supplementary Fig. S1).

We selected seven SGAs that had been prescribed to at least 30 patients in our sample, namely CZP, OZP, ARI, AMI, PAL, RIS, and QUE (Table 1). The choices of these SGAs, which were taken by a substantial number of subjects, allowed more robust models to be constructed. Long-acting injectable and oral formulations were analysed equally, after dose conversion using the standard method [23]. Given that some non-SGA psychotropics and other drugs (e.g. statins, metformin) are commonly prescribed (listed in Supplementary Table S1), and might influence patients’ lipid profiles and BMI, we included these concomitant medications as (time-varying) covariates to account for their dynamic effects throughout the study period, following established approaches in prior studies [24, 25]. As such, we can control for possible confounding due to these drugs. We also included daily drug dosage (mg) of the seven SGAs and treatment duration (month) as random-effect covariates to account for multiple SGA medications, while age, gender and years of education were entered as fixed-effect covariates, similar to previous studies conducted by Pardiñas, Nalmpanti [26] and Adkins, Åberg [12].

**Table 1:** Descriptive statistics of the SGA prescription records in our cohort.

Second Generation Antipsychotics (SGAs)^(1)^	No. of subject with prescription		Total no. of prescriptions		Avg. no. of prescription per subject (SD)	
	TC,LDL,HDL,TG (*N* = 625)	BMI (*N* = 646)	TC,LDL,HDL,TG (*N* = 3 525)	BMI (*N* = 6 640)	TC,LDL,HDL,TG (*N* = 740)	BMI (*N* = 759)
CLOZAPINE	119	125	551	1 045	4.6 (2.8)	8.4 (10.1)
OLANZAPINE	230	307	760	1 447	3.3 (2.6)	4.7 (4.5)
ARIPIPRAZOLE	238	275	728	1 228	3.1 (2.2)	4.5 (4.3)
RISPERIDONE	116	232	209	571	1.8 (1.7)	2.5 (2.5)
AMISULPRIDE	162	216	399	766	2.5 (2.0)	3.5 (3.2)
QUETIAPINE	93	105	173	313	1.9 (1.2)	3 (3.1)
PALIPERIDONE	43	65	95	178	2.2 (1.8)	2.7 (2.3)
Any SGA use	553	567	2 591	5 259	4.7 (2.9)	9.3 (8.8)
Average per SGA	215		688	1 351	N/A	N/A

Remark:

(1) Only the SGA prescribed to more than 30 SCZ patients will be included in the GWAS study.

### Random-effect estimation for SGA-induced lipid/BMI changes

We used linear mixed models (LMMs) to estimate the random effects (random slope) of SGAs on lipid/BMI changes. The random effects quantified how each patient’s lipid/BMI changes deviated from the cohort’s mean, serving as a proxy outcome measure for the severity of the metabolic side effects of each patient. A similar approach has been employed in several PGx GWASs [12, 26, 27]. In addition, we employed an advanced statistical approach to disentangle the within-subject estimates from the between-subject estimates of SGA random effects [28–30]. By focusing on the within-subject effects, we can more accurately estimate the metabolic side effects of SGAs by accounting for unmeasured time-invariant confounders [31].

For each of the seven SGAs, we constructed five random-effect LMMs using lipid profiles (LDL, HDL, TC, TG) and BMI as outcomes. An additional model analysing ‘any SGA use’ was included as a pooled analysis of all seven SGAs, resulting in a total of (8 × 5) 40 models in our primary analyses. Random effect coefficients were extracted from these models for patients prescribed the corresponding SGAs, with detailed specifications provided in Supplementary Text 1. Following an established methodology in longitudinal pharmacogenetics studies [12, 26, 27], these random-effect coefficients served as the primary outcome for subsequent GWAS and MAGMA analyses, with sample sizes varying across models (Table 1). The methodology for identifying optimal random-effect LMM models and estimating within-subject SGA dosages is detailed in Supplementary Text 2 and Supplementary Text 3, respectively [4, 31].

We applied rank-based inverse normal transformation (INT) to the random-effect coefficients [32] to ensure a normal distribution of the outcomes (and residuals) and reduce outlier effects (Supplementary Fig. S3).

### Genome-wide association study (GWAS) analysis

GWAS association tests between SNP dosages and SGA-induced lipid/BMI changes were conducted using PLINK 2.00a [33], with gender and the top ten genetic principal components as covariates. The imputed genotypes were converted to PLINK 2 binary formats to retain dosage information, which can improve the statistical power of the association tests. In our primary analyses, we tested additive genetic models using allelic dosage as the predictor. To capture variants with non-additive genetic effects as advised by Guindo-Martínez, Amela [34], we also performed additional analyses based on dominant, recessive and genotypic (2 degrees of freedom) models, resulting in a total of 120 models for the additional analyses.

For significance thresholds, we considered associations genome-wide (GW) significance, when p-values were less than 5×10^−8^ after Bonferroni correction [35]. Following the approach of Adkins et al. in their GWAS of antipsychotics-induced metabolic side effects [12], we classified associations with false discovery rate (FDR) < 0.2 as “suggestive”. Details of multiple testing correction are presented below.

### MAGMA analyses

We performed gene and gene-set association tests between the imputed genotypes and 40 sets of random-effect coefficients of SGA-induced lipid/BMI changes using MAGMA v1.10 ref. ^35^. To optimize statistical power and sensitivity across various genetic architectures, we built three predefined MAGMA models: (1) principal component regression, (2) the SNP-wise mean, and (3) the SNP-wise top 1. MAGMA then aggregated the resulting gene p-values into a single p-value. Such MAGMA models have been detailed in the MAGMA manual.

### Post-GWAS annotation

LD-clumping was performed using PLINK to identify top SNPs within linkage disequilibrium clusters (with clump-p1 = 5 × 10^−5^, clump-p2 = 0.05, r2 = 0.6 and window size = 250 kb). The top SNPs were annotated using Ensembl Variant Effect Predictor (VEP) v111.0 with VEP cache version 111_GRCh38 [36], including gene information, nearest gene, location, and effect allele frequency in East Asian and European populations. Previous studies reporting GW-significant SNPs within the same genes were annotated based on the GWAS Catalog and Open Target Platform [37, 38]. To uncover potential hidden associations between the identified genes and annotated enriched terms across multiple datasets and resources, integrative gene set enrichment analyses and visualization were conducted using the Enrichr-KG platform [39], incorporating four gene-set libraries: GWAS Catalog (2019) [38], GO biological Process (2021) [40], DisGeNET [41], and Human Phenotype Ontology [42]. For each input gene set, the top five enriched terms per library with an FDR < 0.05 were considered significant. A subnetwork linking the input genes to these enriched terms was visualized using the Enrichr-KG platform.

### Fine-mapping with the SuSiE model

To identify potential causal SNPs, we used the SuSiE fine-mapping approach [43], which reports minimal groups of SNPs (credible set) and calculates posterior inclusion probabilities (PIPs) for causal assessment. We performed fine-mapping ±1 000 kb around each suggestive SNP using an LD reference panel from imputed genotypes. The SuSiE model used SNP p-values, with L = 11 nonzero effects and default parameters. Casual SNPs of the best credible sets were visualized in region plots.

### Multiple testing correction

We employed both Bonferroni correction and FDR approaches for multiple testing correction. We applied Bonferroni correction to account for all 160 GWAS analyses on the same dataset (4 genetic models x 8 SGA categories [including ‘any SGA use’] × 5 metabolic outcomes). The Bonferroni-corrected genome-wide significance threshold is hence *p* = 5 × 10^−8^/160 = 3.125 × 10^−10^. Bonferroni correction controls for the family-wise error rate (probability of any false positives) and is ideal for clinical trials or other studies where false positives must be avoided. However, this method has low statistical power when a large number of hypotheses are tested, for example in genomics studies. Therefore, following a previous PGx study [12], we also implemented FDR correction, which is better suited for exploratory research as it maintains higher statistical power while controlling for false positives at an acceptable level [44].

We calculate FDR separately for each analysis to control the expected proportion of false discoveries among the rejected null hypotheses [45]. Unlike Bonferroni correction, FDR is less sensitive to the number of tests performed as it controls for the *proportion* rather than the absolute number of false discoveries [46, 47]. At our suggestive threshold (FDR < 0.2), on average 80% of significant findings would be expected to be true discoveries. As demonstrated by Efron [47], when FDR is calculated separately for each set of GWAS, the *overall* FDR remains generally controlled, particularly with a large number of tests.

### Power analysis

The power analysis was conducted by extracting effect size estimates from a closely related GWAS by Adkins, Åberg [12]. Their top finding was a SNP in the *MEIS2* gene associated with the effects of risperidone on hip and waist circumference (WC). We focused on the effect size for WC, as it is more relevant to metabolic syndrome [48]. The top SNP, rs1568679, explained 9.93% of the variance in WC, according to the conversion formulae suggested by So, Xue [49].

Using the Genetic Power Calculator developed by Purcell, Cherny [50] and assuming an additive model, we estimated that a sample size of 487 is required to achieve 80% power at a Bonferroni-corrected GW-significance p-value threshold of 3.125 × 10^−10^.

We also calculated power based on FDR, following the method by Liu and Hwang [51]. Here, we assumed a more modest average effect size estimate. Assuming a proportion of 0.90 null markers, and an average SNP heritability of 0.015 among non-null variants, our current sample size of 669 achieves a power of 81.98% at our suggestive FDR threshold of 0.2. Assuming an average SNP heritability of 0.02, the power would reach 92.86%. It is important to note that our longitudinal study design offers a greater effective sample size compared to a cross-sectional approach, which is assumed in the above power calculations.

### Ethical standards and consent to participate

This study adhered to the ethical principles of the Helsinki Declaration and relevant national and institutional guidelines for human research. Ethical approval was obtained from the New Territories West Cluster Ethics Committee (Approval Numbers: NTWC/CREC/823/10 and NTWC/CREC/1293/14) and the Joint Chinese University of Hong Kong-New Territories East Cluster Clinical Research Ethics Committee (Approval Number: 2016.559). All participants provided written informed consent.

## Results

### Sample characteristics

Our final dataset comprised 625 subjects with lipid profile data and 646 subjects with BMI data after the seven SGAs prescribed to at least 30 patients were selected. Supplementary Table S2 presents the gender ratio, mean age at the first clinical visit, and mean years of follow-up. The longitudinal cohort had a maximum follow-up period of 18.7 years, with a mean follow-up of 5.7 years (SD = 3.3) for the lipid cohort and 5.5 years (SD = 3.2) for the BMI cohort (Supplementary Fig. S4). Supplementary Table S3 summarises the number of patients treated with single versus multiple antipsychotics over 3 months within the first 3 years of follow-up.

### GWAS results

#### Primary analyses: Additive genetic model

We conducted 40 separate GWASs to examine the effects of SNPs on SGA-induced changes in lipids (TC, HDL, LDL, TG) and BMI for seven specific SGAs, plus a pooled analysis of any SGA use. Individual GWAS sample sizes ranged from 43 to 567 patients (mean = 215), with mean prescriptions per patient ranging from 1.8 (SD = 1.7) to 9.3 (SD = 8.8) (Table 1).

In our primary GWAS analyses using an additive genetic model, two SNPs reached *p* < 5 × 10^−8^ but did not achieve GW significance after Bonferroni correction for all 160 GWAS models tested (Table 2). An additional eight SNPs met the suggestive threshold of FDR of < 0.2 (Supplementary Table S4). The top SNP, rs6532055 (*p* = 3.13 × 10^−09^, FDR = 0.022), was associated with olanzapine-induced LDL changes. This SNP is located within an intron of the *ABCG2* gene, which is part of the ATP-binding cassette (ABC) family. The second SNP, rs2644520 (*p* = 3.06 × 10^−08^, FDR = 0.122), was associated with aripiprazole-induced TG changes and located in an intergenic region near *SORCS1*, a gene encoding a member of vacuolar protein sorting 10 (VPS10) domain-containing receptor proteins.

**Table 2:** Top five SNPs associated with SGA-induced changes in lipid levels and BMI.

	Primary Analyses		Additional analyses		
SGA	Olanzapine	Aripiprazole	Clozapine	Paliperidone	Quetiapine
Phenotype	LDL	TG	HDL	LDL	TG
Test model^(1)^	Additive	Additive	Genotypic	Genotypic	Dominant
SNP	rs6532055	rs2644520	rs115843863	rs2514895	rs188405603
Gene	*ABCG2*	*-*	*-*	*-*	*SLC2A9*
Nearest	*-*	*SORCS1*	*UPP2*	*KIRREL3*	*-*
Location	Intron	Intergene	Intergene	Intergene	Intron
CHR	4	10	2	11	4
POS (GRCh38)	88197235	105919960	157988744	127240288	9973710
Effect Allele	C	G	T	T	C
N	230	238	119	43	93
Effect AF	0.70	0.44	0.19	0.21	0.07
1KG AF (EAS)	0.73	0.45	0.20	0.29	0.08
1KG AF (EUR)	0.39	0.45	0.03	0.17	0.00
Imp. Rsq	0.73	0.99	0.90	0.97	0.62
Beta	−0.622	0.510	NA	NA	1.641
SE	0.101	0.089	NA	NA	0.269
P-value	3.13E-09	3.06E-08	2.05E-08	4.96E-09	3.54E-08
P-value_Bonferroni_^(2)^	5.01E-07	4.90E-06	3.28E-06	7.94E-07	5.66E-06
FDR	0.022	0.122	0.029	0.004	0.065
λ_GC_ (50, 70, 90 percentiles)	1.00, 1.00, 1.00	1.01, 1.01, 1.01	1.00, 1.01, 1.01	0.97, 0.99, 1.03	1.01, 1.01, 1.01
QQ Plot					

*CHR* chromosome, *POS* position, *SGA* second generation antipsychotics, *SNP* single nucleotide polymorphism, *AF* allele frequency, *1KG* 1000 genomes project, “*Imp. Rsq*” genotype imputation r-squared, *λ*_*GC*_ genomic control inflation factor.

Remarks:

(1) The test model indicates the genetic model employed when conducting GWAS using PLINK2.

(2) P-values are Bonferroni corrected for 4 genetic models across 40 random effects, resulting in a total of 160 GWASs (i.e., p-value x 160).

The quantile-quantile plots (QQ) plots for the GWASs with the two top SNPs are shown at the bottom of Table 2, and QQ plots of the GWASs with SNPs achieving an FDR < 0.2 are shown in Supplementary Table S5. The QQ plots demonstrate that p-value distributions closely match the expected p-values under the null hypothesis, with the genomic control inflation factor (λ_GC_ at the median) ranging from 0.94 to 1.01, indicating that genomic inflation is unlikely to be a concern.

#### Additional analyses with non-additive models

Further analyses using non-additive models (dominant, recessive and genotypic) revealed three additional SNPs with *p* < 5 × 10^−8^. However, none achieved GW significance after Bonferroni correction (Table 2). An additional 17 SNPs with FDRs < 0.2 were identified under non-additive models (Supplementary Table S6). The top SNP in the genotypic model, rs115843863 (*p* = 2.05 × 10^−8^, FDR = 0.0287), was associated with clozapine-induced HDL changes. The SNP is located in an intergenic region near *UPP2*, a gene involved in dCMP and uridine catabolic processes. Another SNP in the genotypic model, rs2514895 (*p* = 4.96 × 10^−9^, FDR = 0.004), was associated with paliperidone-induced LDL changes and is located near *KIRREL3*, a gene encoding a nephrin-like protein expressed in the brain. The last SNP rs188405603 (*p* = 3.52 × 10^−8^, FDR = 0.065) under the dominant model was associated with quetiapine-induced TG changes and is located within an intron of *SLC2A9*, a gene encoding a glucose transporter.

#### Suggestive associations with FDR < 0.2

Eight SNPs achieved an FDR < 0.2 in the primary GWAS analyses under the additive model (Supplementary Table S4). Notably, four SNPs, namely rs7412 (FDR = 0.182), rs2384157 (FDR = 0.195), rs74625905 (FDR = 0.195) and rs56349742 (FDR = 0.195), were associated with olanzapine-induced LDL changes. The well-known LDL-altering SNP rs7412 in *APOE* is positively associated with olanzapine-induced LDL changes. Four additional SNPs were associated with quetiapine-induced HDL changes, namely rs2358259 (FDR = 0.123), rs10174314 (FDR = 0.123), rs117416034 (FDR = 0.123), and rs6424242 (FDR = 0.186). In particular, rs6424242 is located in an upstream region of the *SIPA1L2* gene, which has been previously linked to obesity-related traits, response to alcohol consumption, and neuroticism based on Open Targets and the GWAS Catalog [52–54].

In the additional GWAS analyses under non-additive models, 17 SNPs with FDRs < 0.2 were identified. These SNPs were associated with metabolic side effects of clozapine, olanzapine, risperidone, and paliperidone (Supplementary Table S6). The associated genes have known implications in psychiatric disorders, lipid or BMI measurements, or drug responses, including *BICD1* and *CSMD1* (olanzapine-induced TC changes); *GADL1* (risperidone-induced BMI changes); *SIPA1L2* (quetiapine-induced HDL changes); and *RAB38*, *CDH23*, *AMPH*, *FOXN3*, *APBB2*, *C1R* and *LRCOL1* (paliperidone-induced LDL changes). Table 3 provides a comprehensive overview of all identified genes from different analyses.

**Table 3:**
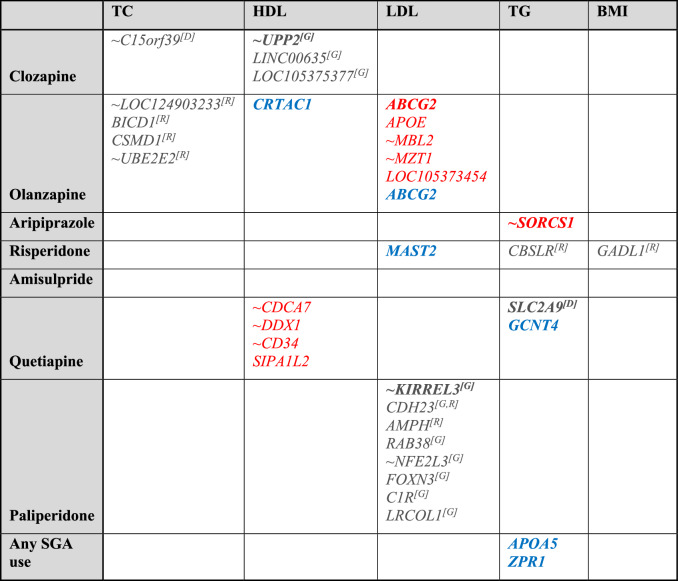
Genes associated with SGA-induced lipid/BMI changes across various analyses^(1,2,3,4)^.

Remarks:

(1) Gene in  was identified by the primary analysis, gene in black was identified by the additional analysis, gene in  was identified by the MAGMA analysis.

(2) **Bold text**: SNP-associated gene (SNP’s *p* < 5 × 10^−8^ before Bonferroni correction)/Gene reaching GW significant threshold (*p* < 2.73 × 10^−6^), Normal text: SNP-associated gene/Gene reaching the suggestive evidence threshold (FDR < 0.2).

(3) ~Gene: SNP does not fall within the gene, but it is located near the gene.

(4) Superscript *[D]* Dominant, *[R]* Recessive *[G]* genotypic: indicates the genetic model being used in the GWAS analysis.

As emphasised earlier, we caution that none of the findings passed Bonferroni correction. The above results (with FDR < 0.2) should be considered tentative and further replications are required to confirm our findings.

#### Fine-mapping results

The fine-mapping results for the top five SNPs are visualized in region plots (Supplementary Table S7). The top SNPs associated with olanzapine-induced LDL changes and aripiprazole-induced TG changes were proposed to be causal (PIP = 1.0). However, the remaining three top SNPs were not considered causal, as shown by their low PIP values. Another SNP, rs73968514 (PIP = 1.0), was identified as potentially causal for clozapine-induced HDL changes (PIP = 1.0), replacing the original GWAS hit rs115843863. Both rs2441693 and another SNP rs2441693, with the same p-value, were identified as potentially causal for paliperidone-induced LDL effects (PIP = 0.5 each). Finally, instead of the observed GWAS hit rs188405603, fine-mapping evidence suggested that rs77140241 was the real causal variant for quetiapine-induced TG changes (PIP = 1.0, *p* = 9.5 × 10^−8^, FDR = 0.065).

### MAGMA analysis results

#### Gene-level analysis

Six genes reached the GW-significance p-value threshold of 2.73 × 10^−6^ after Bonferroni correction (α = 0.05/18288 genes tested) in the gene-level analysis (Table 4), with their corresponding QQ plots from the gene-level analysis shown in Supplementary Table S8. All GW-significant genes also had an FDR < 0.05. Diseases or traits associated with these genes were annotated using the Open Target Platform [37], which we also highlighted here. The top gene *ABCG2* (*p* = 8.26 × 10^−9^, FDR = 1.51 × 10^−4^) was associated with olanzapine-induced LDL changes; this gene is related to gout, urate measurement, and BMI based on information from the Open Target Platform. *APOA5* (*p* = 3.45 × 10^−8^, FDR = 6.31 × 10^−4^) and *ZPR1* (*p* = 1.80 × 10^−6^, FDR = 0.016) were associated with SGA-induced TG changes; these genes were related to TG, HDL, and LDL levels and metabolic syndrome. *GCNT4* (*p* = 3.17 × 10^−7^, FDR = 5.12 × 10^−3^) and *MAST2* (*p* = 4.79 × 10^−7^, FDR = 8.62 × 10^−3^) were associated with quetiapine-induced TG and risperidone-induced LDL changes respectively. *CRTAC1* (*p* = 2.273 × 10^−6^, FDR = 0.042) was associated with olanzapine-induced HDL changes. Based on the evidence from the Open Target Platform [37], *GCNT4* and *MAST2* are related to neurodegenerative disease and measurements of erythrocyte count, BMI, LDL and TC [55–59]; whereas *CRTAC1* is related to body fat percentage and measurements of HDL and TG [59, 60].

**Table 4:** Significant gene associations with SGA-induced changes in lipid levels and BMI identified in MAGMA gene-level analyses (*p* < 2.73 × 10^−6^)^(1)^.

SGA	Trait	Gene	P-value^(2)^	FDR	λ_GC_	Associated diseases (association scores: 0–1) from the Open Target Platform^(3)^
Olanzapine	LDL	*ABCG2*	8.26E-09	1.51E-04	0.907	Gout(0.92), urate measurement(0.91), BMI(0.83), drug use measurement(0.76), neuroimaging measurment(0.76)
Any SGA use	TG	*APOA5*	3.45E-08	6.31E-04	0.925	TG measurement(0.77), HDL measurement(0.71)
Quetiapine	TG	*GCNT4*	3.17E-07	5.13E-03	0.961	Neurofibrillary tangles measurement(0.47), BMI(0.46), LDL measurement(0.46), TC measurement(0.45)
Risperidone	LDL	*MAST2*	4.79E-07	8.62E-03	0.934	Erythrocyte count(0.46), BMI(0.34), neurodegenerative disease(0.28)
Any SGA use	TG	*ZPR1*	1.80E-06	1.64E-02	0.925	TG measurement(0.82), HDL measurement(0.67), LDL measurement(0.67), metabolic syndrome(0.59)
Olanzapine	HDL	*CRTAC1*	2.27E-06	4.15E-02	0.934	Body fat percentage(0.82), HDL measurement(0.79), TG measurement(0.74)

*λ*_*GC*_ Genomic control inflation factor.

Remarks:

(1) The total number of genes involved in the MAGMA gene-level analysis is 18 288.

(2) The significant p-value threshold after Bonferroni correction for multiple testing is 2.73 × 10^−6^ ( = 0.05/18288).

(3) Associated disease scores were extracted from the genetic association column in the Open Target Platform.

**Fig. 1:**
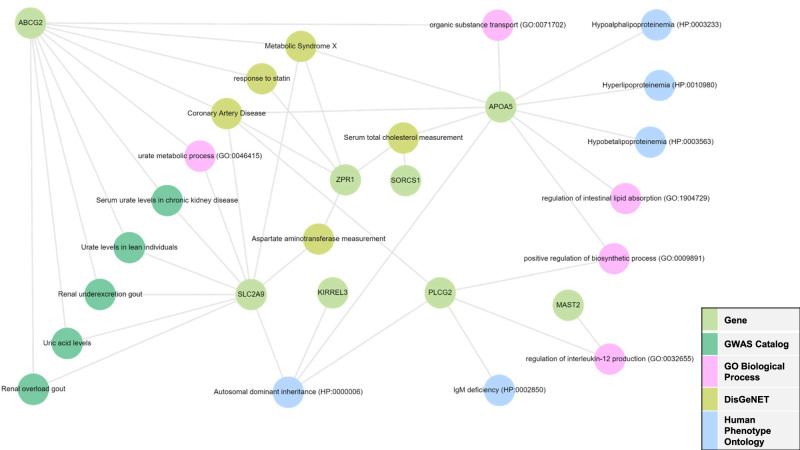
Subnetwork of top genes linked to enriched terms. This subnetwork illustrates the top five enriched terms (FDRs < 0.05) for each library, connected to the 11 genes highly associated with the SGA-induced metabolic side effects, as identified through the GWAS and gene-level MAGMA analyses.

Gene set enrichment analysis, incorporating these six significant genes along with those associated with the top five SNPs identified in the GWAS analyses, was performed using the Enrichr-KG platform [39]. The subnetwork of gene and enriched terms are illustrated in Fig. 1, with corresponding enrichment p-values and FDRs listed in Supplementary Table S9.

#### Gene set analysis

Fourteen gene sets were nominally associated with SGA-induced metabolic changes. After FDR correction (FDR < 0.05), four gene sets remained significant (Supplementary Table S10). The top gene set, *skeletal muscle satellite cell differentiation* (p_Bonferroni _= 1.29 × 10^−5^, FDR = 4.4 × 10^−4^), was associated with SGA-induced TG changes [61]. The *mRNA editing* (p_Bonferroni_ = 2.20 × 10^−5^, FDR = 4.4 × 10^−4^) gene set was associated with clozapine-induced LDL changes. The gene sets *ER ubiquitin ligase complex* (p_Bonferroni _= 0.004, FDR = 0.04) and *Saccadic smooth pursuit* (p_Bonferroni _= 0.004, FDR = 0.04) were associated with clozapine-induced BMI and amisulpride-induced BMI changes respectively.

## Discussion

This study represents one of the largest longitudinal PGx GWAS investigations, identifying genetic variants associated with lipid and BMI changes induced by seven commonly used SGAs in a Chinese SCZ cohort. Our investigation included 19 316 prescription records and 3 917 to 7 596 metabolic measurements for each outcome, with a median follow-up duration of 5.7 years (SD = 3.3, max = 18.7), surpassing the duration of comparable GWASs [12,14–16].

Our study design incorporates several key strengths. Notably, our cohort recruited from an early psychosis intervention clinic comprised a high proportion of antipsychotic-naïve patients (approximately 63%) at baseline; as such, confounding by previous medications was reduced and likely lower than many other comparable studies, including Adkins et al. [12]. Our focus on a homogeneous ethnic Chinese sample provides valuable insights specific to this underrepresented population, particularly important given the known differences in allele frequency and LD patterns between East Asian and European populations [62–65], as evidenced in Table 2.

We employed a sophisticated analytical approach using within-subject random effects of SGA-induced lipid/BMI changes. This method substantially reduces the risk of confounding by indication/contraindication [66]. To further mitigate potential confounding effects, we included lipid-lowering drugs as covariates in the GWAS phenotype estimations. The mean age of our cohort at the first clinical visit (28.3 years, SD = 9.8) was lower than that reported in a similar study [12], reducing the influence of age-related metabolic changes on our findings.

The top SNP rs6532055 is located in *ABCG2*, which encodes a translocation protein involved in the efflux of antipsychotics across cellular membranes [67, 68]. Its association with olanzapine-induced LDL changes suggests a potential role in antipsychotic pharmacokinetics and lipid metabolism. Notably, *ABCG2* has also been associated with LDL reduction in response to rosuvastatin [69, 70].

Another top gene identified was *SORCS1* which was associated with aripiprazole-induced triglyceride changes. *SORCS1* encodes a member of the VPS10 domain-containing receptor protein family and is strongly expressed in the central nervous system [71]. It has been implicated in insulin regulation and type 2 diabetes risk in both animal and clinical studies [72–74]. Notably, several studies have shown that increased TG levels are associated with increased type 2 diabetes risk and impaired fasting glucose [75–77]. Its role in energy balance further supports its potential involvement in antipsychotic-induced metabolic alterations [78].

*UPP2*, linked to clozapine-induced HDL changes, encodes uridine phosphorylase 2. Several studies have revealed an association between uridine metabolism with lipid metabolism and glucose homeostasis [79–81]. Increasing endogenous hepatic uridine levels by inhibiting uridine phosphorylase 2 may reduce drug-induced liver lipid accumulation [81, 82], although long-term uridine consumption might promote liver lipid accumulation and exacerbate glucose intolerance [81].

*KIRREL3*, linked with paliperidone-induced LDL changes, encodes a synaptic cell adhesion molecule essential for the formation of target-specific synapses and is expressed in fetal and adult brain tissues. While its role in lipid metabolism remains to be investigated, this finding suggests a potential novel link between neuronal function and metabolic regulation.

*SLC2A9*, linked to TG changes in our sample, encodes glucose transporter 9 (GLUT9), a protein involved in reabsorbing or excreting urate and glucose in kidney proximal tubules. This gene has been strongly associated with uric acid levels and gout in numerous studies [83–86]. Studies have revealed a significant positive association between TG and urate levels [87–89], and a recent GWAS from Qatar revealed the association of *SLC2A9* with LDL levels [90].

We caution that these top five SNPs did not achieve GW significance after Bonferroni correction. However, these preliminary findings may indicate potential targets for future investigation on the biological mechanisms of antipsychotic-induced metabolic effects and may inform personalised prescription strategies.

Our primary analyses under an additive model identified eight additional SNPs with suggestive evidence (FDR < 0.2), located in or near *APOE*, *MBL2*, *MZT1*, *LOC105373454*, *CDCA7*, *DDX1*, *CD34*, and *SIPA1L2* (Supplementary Table S4). Many of these genes are associated with lipid levels, diabetes, CVD, urate levels, or other metabolic measurements based on data from the Open Target Platform [37]. Similar evidence was found for 17 suggestive SNPs (FDR < 0.2) identified in our additional analyses using non-additive genetic models (Supplementary Table S6).

MAGMA gene-level analyses identified six GW-significant genes associated with SGA-induced lipid/BMI changes (Table 4). Notably, *ABCG2* was identified via both GWAS and MAGMA analyses, providing further support for its potential role in olanzapine-induced LDL changes. In analyses of patients taking any of the seven SGAs, we identified two genes, *APOA5* and *ZPR1*, which are significantly associated with SGA-induced TG changes. These findings are consistent with previous research. *APOA5* encodes apolipoprotein A5 (apoA5), a protein that regulates plasma TG levels through enhancing the catabolism of TG-rich lipoproteins and inhibiting very-low-density lipoprotein (VLDL) production [91]. This gene has been strongly associated with TG, HDL, LDL and metabolic syndrome [92–94]. *ZPR1*, located near the apolipoprotein gene cluster *APOA1*/*C3*/*A4*/*A5*, encodes a regulatory protein that binds various transcription factors and interacts with *APOA5* [95]. Similar to *APOA5*, *ZPR1* regulates TC, HDL and TG levels and has been associated with hypertriglyceridemia, metabolic syndrome and type 2 diabetes mellitus [91, 96–98]. *APOA5* and *ZPR1* may represent shared genetic mechanisms underlying metabolic side effects across different SGAs.

Our study has several limitations. First, only seven SGAs were included, although these are probably among the most commonly prescribed. Future research should aim to expand the scope to include a broader range of SGAs. Second, while our sample sizes are relatively large compared to similar GWASs [13–16, 99] (and among the largest for GWAS on SGA-induced metabolic side-effects over a medium to long term), power analysis indicated that our GWAS models may be underpowered to achieve the stringent Bonferroni-corrected GW-significance p-value threshold of 3.125 × 10^−10^, particularly for analyses involving specific SGAs with smaller sample sizes. Therefore, caution should be exercised when extrapolating conclusions from these analyses. A larger cohort in future studies would enhance the statistical power and robustness of our findings. Third, potential residual confounding may affect the estimation of the metabolic side effects of SGAs, which may in turn affect the estimation of the genetic influence on these side effects. Although we have applied sophisticated methods and controlled for concomitant and multiple SGA medications, there may be unmeasured confounders that could impact our results. In addition, our methods cannot account for historical treatment effects of SGAs which have been prescribed to patients before recruitment. However, our longitudinal design, which involves a relatively long follow-up period, may be less affected by the effects of prior medications compared to short-term studies. The effects of prior drugs are possibly ‘diluted’ over a long follow-up. Finally, lifestyle factors such as diet, exercise, alcohol consumption and tobacco smoking were not measured and may be included in future studies.

Despite these limitations, our study provides valuable insights into the pharmacogenetics of SGA-induced metabolic changes in a Chinese SCZ cohort. The identified genetic markers not only enhance our understanding of the biological mechanisms underlying these metabolic changes but also hold promise for developing more tailored and safer treatment strategies for individuals with SCZ. However, further studies and replication are needed before these genetic findings can be applied in clinical practice.

## Supplementary information

Supplementary Table S1-S10

Supplementary Text 1-4 and Supplementary Fig. S1-S5

## Data Availability

The data that support the findings of this study are available upon request from the corresponding authors, Hon-Cheong So and Simon Sai-Yu Lui. The data is not publicly available because it contains information that could compromise the privacy of research participants. The software and tools used in this study are listed in Supplementary Text 4.
