# Supplementary material for: Pharmacogenetic study of antipsychotic–induced lipid and BMI changes in Chinese schizophrenia patients: A Genome-Wide Association Study
Source: Transl Psychiatry. 2025 Aug 19;15:295. doi: 10.1038/s41398-025-03499-w (PMC12365156; doi:10.1038/s41398-025-03499-w)
Supplement: Supplementary file 2 — Supplementary Text 1-4 and Supplementary Fig. S1-S5 [file 41398_2025_3499_MOESM2_ESM.docx]

## Supplementary Text 1: The full formula of 40 random-effect linear mixed models (LMMs) expressed in lmer4 (R package) format.

**Random-effect LMM model for CLOZAPINE (one model per outcome)**

Log(TC/HDL/LDL/TG/BMI measure) ~ age + gender + education + tx_duration +

dose.OLANZAPINE + dose.ARIPIPRAZOLE + dose.RISPERIDONE + dose.AMISULPRIDE + dose.QUETIAPINE + dose.PALIPERIDONE +

dose.HALOPERIDOL + dose.TRIFLUOPERAZINE + dose.VALPROATE + dose.LITHIUM + dose.CITALOPRAM + dose.SERTRALINE +

dose.METFORMIN + dose.ATORVASTATIN + dose.SIMVASTATIN +

**bz_dose.CLOZAPINE + wz_dose.CLOZAPINE + (1 + wz_dose.CLOZAPINE | ID)**

**Random-effect LMM model for OLANZAPINE (one model per outcome)**

Log(TC/HDL/LDL/TG/BMI measure) ~ age + gender + education + tx_duration +

dose.CLOZAPINE + dose.ARIPIPRAZOLE + dose.RISPERIDONE + dose.AMISULPRIDE + dose.QUETIAPINE + dose.PALIPERIDONE +

dose.HALOPERIDOL + dose.TRIFLUOPERAZINE + dose.VALPROATE + dose.LITHIUM + dose.CITALOPRAM + dose.SERTRALINE +

dose.METFORMIN + dose.ATORVASTATIN + dose.SIMVASTATIN +

**bz_dose.OLANZAPINE + wz_dose.OLANZAPINE + (1 + wz_dose.OLANZAPINE | ID)**

**Random-effect LMM model for ARIPIPRAZOLE (one model per outcome)**

Log(TC/HDL/LDL/TG/BMI measure) ~ age + gender + education + tx_duration +

dose.CLOZAPINE + dose.OLANZAPINE + dose.RISPERIDONE + dose.AMISULPRIDE + dose.QUETIAPINE + dose.PALIPERIDONE +

dose.HALOPERIDOL + dose.TRIFLUOPERAZINE + dose.VALPROATE + dose.LITHIUM + dose.CITALOPRAM + dose.SERTRALINE +

dose.METFORMIN + dose.ATORVASTATIN + dose.SIMVASTATIN +

**bz_dose.ARIPIPRAZOLE + wz_dose.ARIPIPRAZOLE + (1 + wz_dose.ARIPIPRAZOLE | ID)**

**Random-effect LMM model for RISPERIDONE (one model per outcome)**

Log(TC/HDL/LDL/TG/BMI measure) ~ age + gender + education + tx_duration +

dose.CLOZAPINE + dose.OLANZAPINE + dose.ARIPIPRAZOLE + dose.AMISULPRIDE + dose.QUETIAPINE + dose.PALIPERIDONE +

dose.HALOPERIDOL + dose.TRIFLUOPERAZINE + dose.VALPROATE + dose.LITHIUM + dose.CITALOPRAM + dose.SERTRALINE +

dose.METFORMIN + dose.ATORVASTATIN + dose.SIMVASTATIN +

**bz_dose.RISPERIDONE + wz_dose.RISPERIDONE + (1 + wz_dose.RISPERIDONE | ID)**

**Random-effect LMM model for AMISULPRIDE (one model per outcome)**

Log(TC/HDL/LDL/TG/BMI measure) ~ age + gender + education + tx_duration +

dose.CLOZAPINE + dose.OLANZAPINE + dose.ARIPIPRAZOLE + dose.RISPERIDONE + dose.QUETIAPINE + dose.PALIPERIDONE +

dose.HALOPERIDOL + dose.TRIFLUOPERAZINE + dose.VALPROATE + dose.LITHIUM + dose.CITALOPRAM + dose.SERTRALINE +

dose.METFORMIN + dose.ATORVASTATIN + dose.SIMVASTATIN +

**bz_dose.AMISULPRIDE + wz_dose.AMISULPRIDE + (1 + wz_dose.AMISULPRIDE | ID)**

**Random-effect LMM model for QUETIAPINE (one model per outcome)**

Log(TC/HDL/LDL/TG/BMI measure) ~ age + gender + education + tx_duration +

dose.CLOZAPINE + dose.OLANZAPINE + dose.ARIPIPRAZOLE + dose.RISPERIDONE + dose.AMISULPRIDE + dose.PALIPERIDONE +

dose.HALOPERIDOL + dose.TRIFLUOPERAZINE + dose.VALPROATE + dose.LITHIUM + dose.CITALOPRAM + dose.SERTRALINE +

dose.METFORMIN + dose.ATORVASTATIN + dose.SIMVASTATIN +

**bz_dose.QUETIAPINE + wz_dose.QUETIAPINE + (1 + wz_dose.QUETIAPINE | ID)**

**Random-effect LMM model for PALIPERIDONE (one model per outcome)**

Log(TC/HDL/LDL/TG/BMI measure) ~ age + gender + education + tx_duration +

dose.CLOZAPINE + dose.OLANZAPINE + dose.ARIPIPRAZOLE + dose.RISPERIDONE + dose.AMISULPRIDE + dose.QUETIAPINE +

dose.HALOPERIDOL + dose.TRIFLUOPERAZINE + dose.VALPROATE + dose.LITHIUM + dose.CITALOPRAM + dose.SERTRALINE +

dose.METFORMIN + dose.ATORVASTATIN + dose.SIMVASTATIN +

**bz_dose.PALIPERIDONE + wz_dose.PALIPERIDONE + (1 + wz_dose.PALIPERIDONE | ID)**

The dose of the drug (*dose.<drug>*) represents the time-varying covariate of second-generation antipsychotics (SGAs), first-generation antipsychotics (FGA), and other psychotropic or concomitant drug daily dose (mg) that may affect the lipid/BMI measure. The between-subject and within-subject target SGA doses (mg) are denoted by *bz_dose.<target SGA>* and *wz_dose.<target SGA>,* respectively. These dose-related covariates were recorded 21 days before the lipid or BMI measurements.

The subject identifier (*ID*) serves as the grouping factor, denoted by (……|ID). This indicates that the model is a random-effect (also known as random-slope) linear mixed-effects model (LMM). The random-effect coefficients of *wz_dose.<target SGA>* will be extracted from the fitted model, representing the phenotype in our GWAS and MAGMA analyses.

**Random-effect LMM model for SGAs - binary variable (one model per outcome)**

Log(TC/HDL/LDL/TG/BMI measure) ~ age + gender + education + tx_duration +

FGA + VALPROATE + LITHIUM + CITALOPRAM + SERTRALINE +

METFORMIN + ATORVASTATIN + SIMVASTATIN +

**bz_SGA + wz_SGA + (1 + wz_SGA | ID)**

The binary variable for the drug *(<drug>*) represents the time-varying binary covariate of SGAs, first-generation antipsychotics (FGA), and other psychotropic or concomitant drug daily prescription that may affect the lipid/BMI measure (0 – not prescribed, 1 - prescribed). The between-subject and within-subject SGA binary variables are denoted by *bz_SGA* and *wz_SGA*, respectively. These binary covariates were recorded 21 days before the lipid or BMI measurements.

The subject identifier (*ID*) serves as the grouping factor, denoted by (……|ID). This indicates that the model is a random-effect (also known as random-slope) LMM. The random-effect coefficients of *wz_SGA* will be extracted from the fitted model, representing the phenotype in our GWAS and MAGMA analyses.

## Supplementary Text 2: Selecting the best model based on the “Side Effect Latency Period”.

We hypothesized that the metabolic side effects induced by SGAs would require a certain amount of time to manifest. To account for this hypothesis, the “side effect latency period” was defined as the interval between the initiation of SGA treatment and the observed changes in the outcome variables. Linear mixed models (LMMs) were fitted using varying latency periods (2, 4, 7, 14, 21, 28, and 35 days) to identify the optimal model based on the lowest Akaike Information Criterion (AIC). The AIC values for each model were summarized and plotted against the latency period, as shown in **Supplementary Fig. S5**. For total cholesterol (TC) and high-density lipoprotein (HDL) levels, the optimal latency period ranged from 7 to 35 days. Among these, models with a 21-day latency period consistently demonstrated near-minimal AIC values, and this latency period was chosen as the standard for subsequent analyses in this study.

## Supplementary Text 3: Estimating the within-subject random effects of SGAs on outcome variables.

To differentiate the between-subject and within-subject effects of SGAs on the outcome variables, we applied a hybrid linear mixed model (LMM)^1^. This approach allowed us to examine the association between time-varying predictors and outcomes, while separating the within-subject effect (i.e., changes over time within an individual) from the between-subject effect (i.e., differences between individuals). The between-subject component was modelled using the subject’s expected value of the predictor (e.g., mean SGA dosage, or expected dosage from a regression model), while the within-subject component represented deviations of the predictor from the individual’s expected value at each time point (e.g., detrended SGA dosage can be used as the within-subject component). This method, as described by Curran and Bauer ^2^, accounts for potential confounding by indication or contraindication.

The hybrid LMM was structured to incorporate the optimal latency period of 21 days. Specifically, the SGA dose taken 21 days prior to a given time point by subject *i* (denoted as ${dose.CLOZAPINE}_{\left( t-21 \right), i}$) was used as the time-varying predictor, rather than the dosage at the time of measurement ( ${dose.CLOZAPINE}_{t, i}$). The model included two components: the between-subject variable ( ${bz\_dose.CLOZAPINE}_{i}$) and the within-subject variable ( ${wz\_dose.CLOZAPINE}_{\left( t-21 \right), i}$ ). These components were incorporated into the hybrid LMM as follows:

${log(BMI}_{ti})$ = $\alpha+\beta_{bz}$ ∙${bz\_dose.CLOZAPINE}_{i}$ + $\beta_{wz}$∙ ${wz\_dose.CLOZAPINE}_{\left( t-21 \right), i}$ + …. + ($U_{i}+R_{ti}$)

Here, $U_{i}$ represents the random effect for each subject, and $R_{ti}$ is the residual error specific to time and subject. The time-varying predictor ${dose.CLOZAPINE}_{\left( t-21 \right), i}$was decomposed into its between-subject ${bz\_dose.CLOZAPINE}_{i}$ and within-subject ${wz\_dose.CLOZAPINE}_{\left( t-21 \right), i}$ components through the following regression:

${dose.CLOZAPINE}_{\left( t-21 \right), i}$= $\beta_{0i}+ \beta_{1i}\cdot{tx\_duration}_{ti}+e_{ti}$

In this equation, ${tx\_duration}_{ti}$ refers to the duration of SGA treatment at time *t* for subject *i,* and $e_{ti}$ reflects the residual deviation from the subject’s expected value at time *t*. When no medication had been administered before the first metabolic measurement (${tx\_duration}_{ti}$ = 0), the value of ${dose.CLOZAPINE}_{\left( t-21 \right), i}$ was set to zero. The intercept ($\beta_{0i}$) represents the subject-specific average dose at baseline (*t* = 0), which corresponds to the between-subject variable ( ${bz\_dose.CLOZAPINE}_{i}$). The residual term ($e_{ti}$) captures the detrended, time-specific deviations of the dose and represents the within-subject estimate (${wz\_dose.CLOZAPINE}_{\left( t-21 \right), i}$ ). Once we get within-subject estimate of a dose (${wz\_dose.CLOZAPINE}_{\left( t-21 \right), i}$ ), we can evaluate the within-subject random effect of SGA on the outcome variable (i.e. the GWAS phenotype) from the LMMs specified in the above Supplementary Text 1.

By decomposing the predictor into these components, the hybrid LMM effectively accounted for individual variability and allowed for robust estimation of the within-subject effects of SGAs on metabolic outcomes. Additionally, this model accommodated imbalanced longitudinal data, including irregular time points and missing values, making it well-suited for the complex nature of this study.

## Supplementary Text 4: Software and tools used in this project.

| **Procedures** | **Tools** |
| --- | --- |
| Quality control of genotype | PLINK v1.9p |
| Liftover from GRCh37 to GRCh38 | CrossMap v0.6.4 |
| Genotype imputation | Eagle2,  Minimac4,  ChinaMAP phase1.v1 reference panel |
| Random-effect modelling | R script run on Rstudio v1.4.1106 and R v4.05 with the following R packages: • data.table v1.14.0  • dplyr v1.0.6 • lme4 v1.1.27  • lubridate v1.7.10 • zoo v1.8.9 |
| GWAS analyses | PLINK v2.00a |
| Fine mapping | SusieR v0.12.41 |
| Gene-based and gene-set analyses | MAGMA v1.10 |
| Post-GWAS annotation | VEP v111.0 with VEP cache v111_GRCh38. |
|  | Open Target Platform |
|  | GWAS Catalogue |
| FDR estimation | R package: qvalue v2.15.0 |
| Power analysis | Genetic Power Calculator^3^ |

## Supplementary Fig. S1: Distribution of lipid and BMI measures before and after natural log transformation.

Natural log transformation was applied on the lipid/BMI measures to better satisfy the normality assumptions underlying our linear mixed models. The upper half shows the measures before transformation, while the lower half shows the measures after transformation.

## Supplementary Fig. S2: Detection of ethnic outliers using multidimensional scaling (MDS).


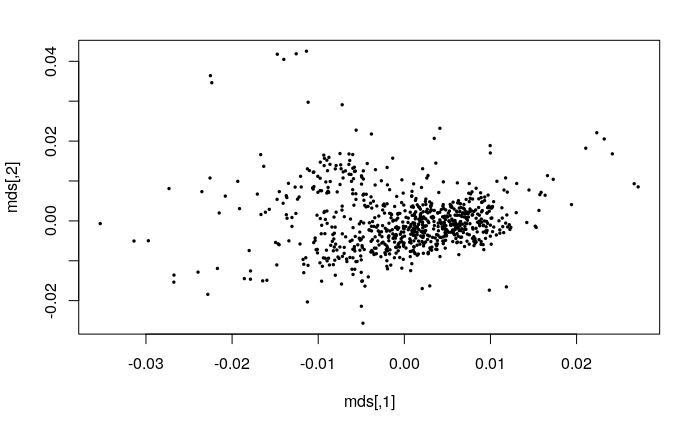


No significant outliers were identified.

##
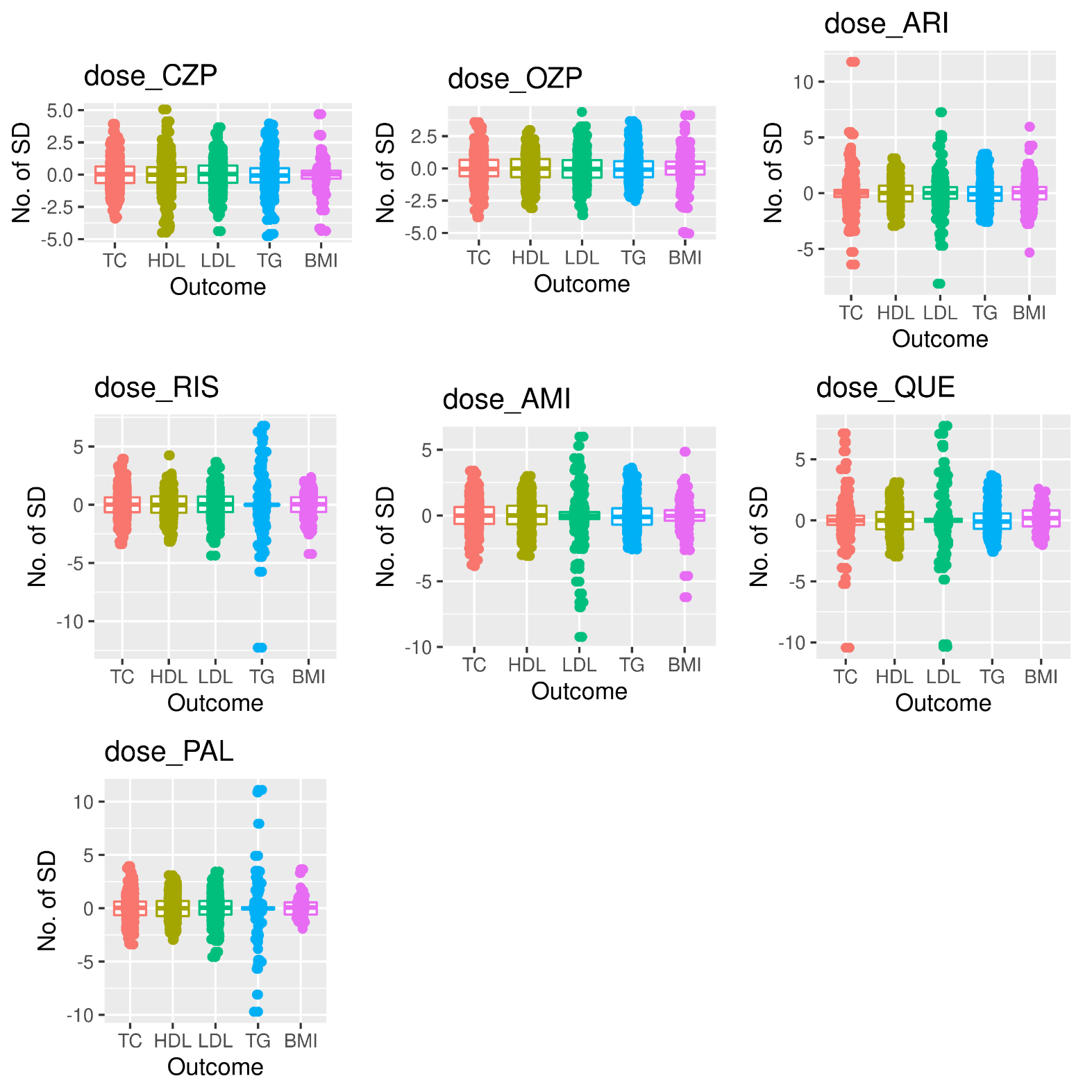
Supplementary Fig. S3: The distribution of random-effect coefficients (GWAS phenotypes) showing the existence of strong outliers.

## Supplementary Fig. S4: The histogram of the treatment duration of patients in lipid and BMI cohort.


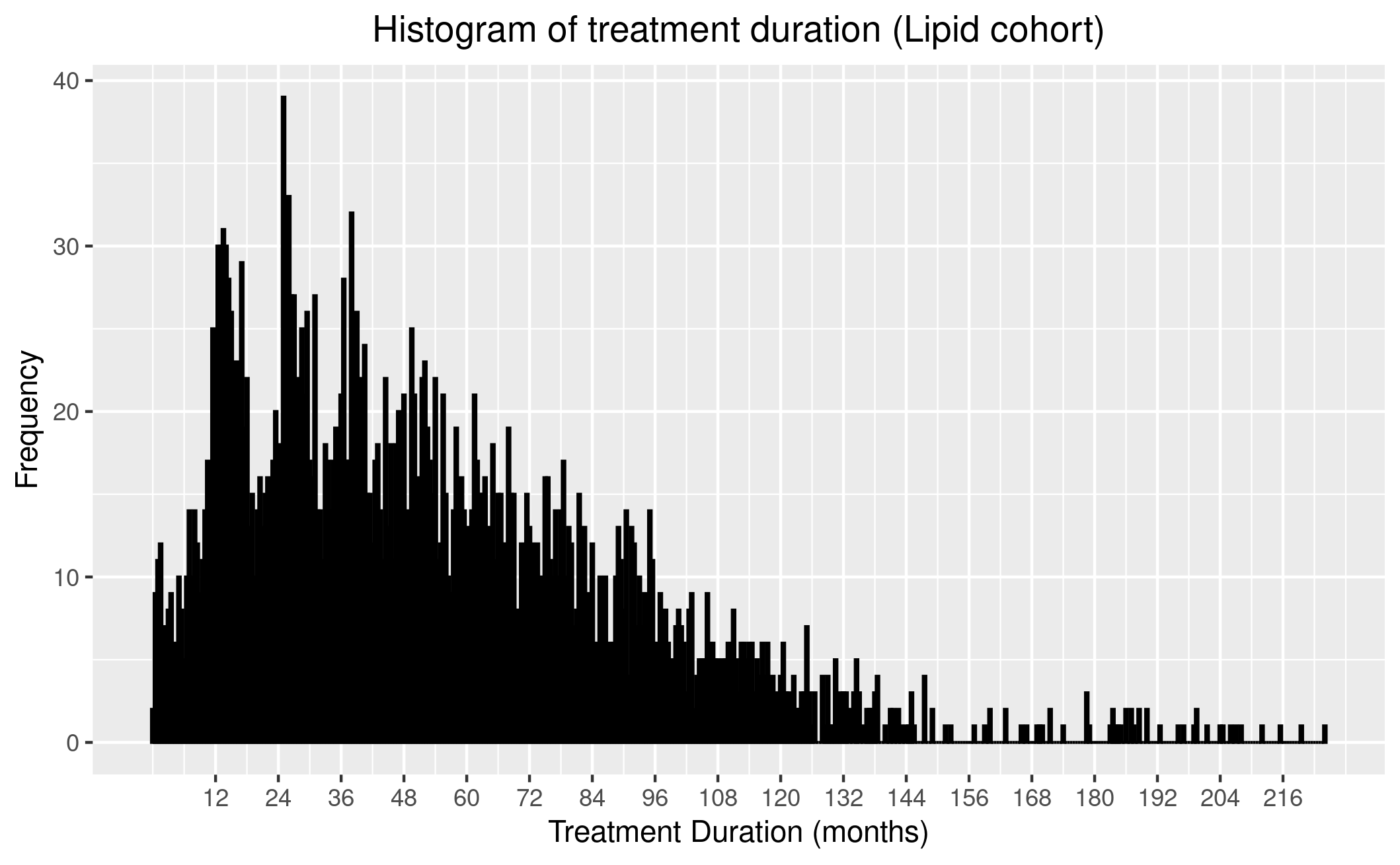


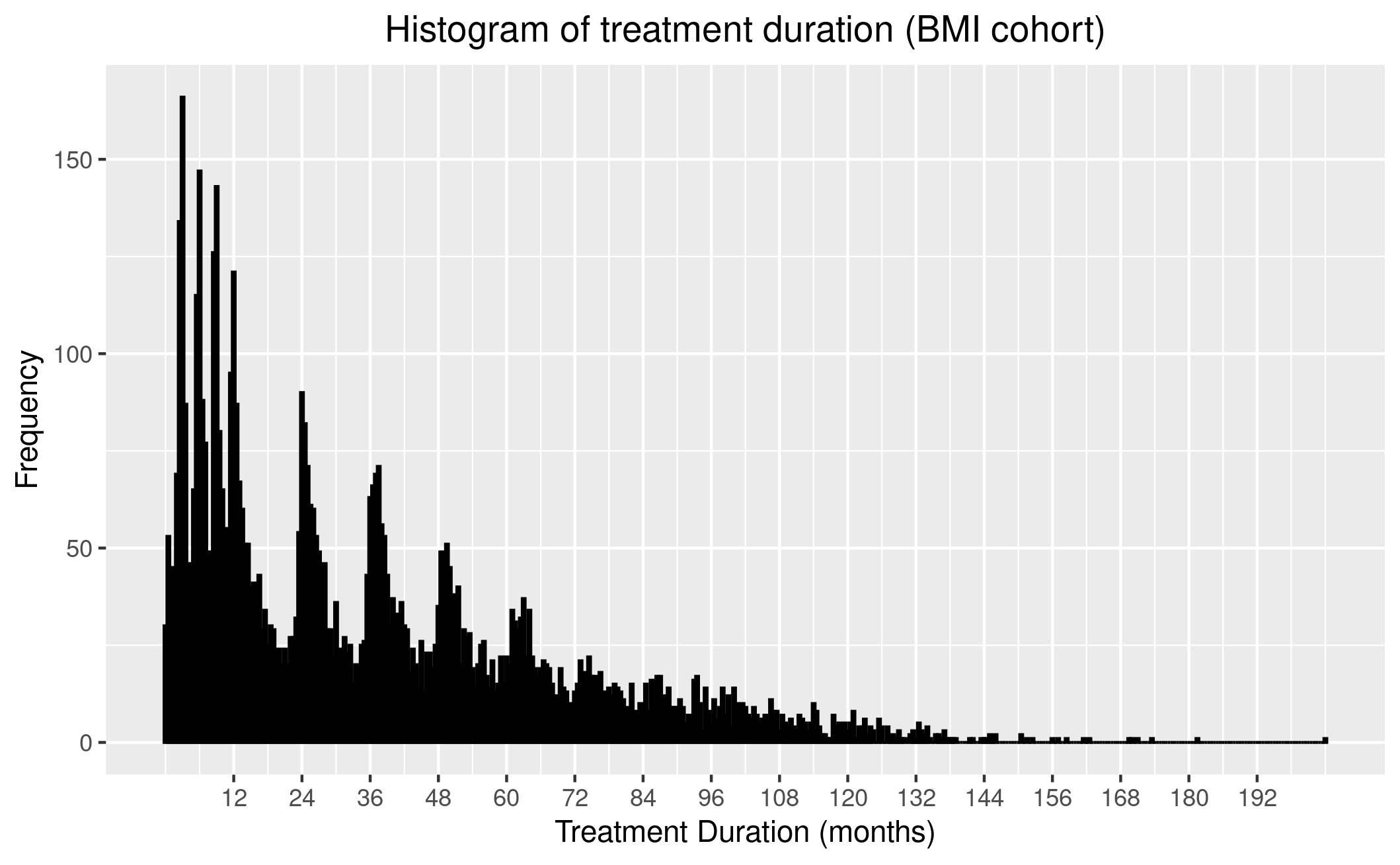


## Note: Lipid and BMI measurements for all patients were taken at the 0th month, with frequencies of 625 and 646, respectively. These records were excluded from the plots to improve the scaling of the y-axis.

## Supplementary Fig. S5: Models’ AIC against the response time of SGA-induced metabolic side effects^4^.

Note: Models with lower AIC values are preferred over models with higher AIC values.

## References:

1. Twisk, J.W.R. & de Vente, W. Hybrid models were found to be very elegant to disentangle longitudinal within- and between-subject relationships. *Journal of Clinical Epidemiology* **107**, 66-70 (2019).

2. Curran, P.J. & Bauer, D.J. The disaggregation of within-person and between-person effects in longitudinal models of change. *Annual review of psychology* **62**, 583 (2011).

3. Purcell, S., Cherny, S.S. & Sham, P.C. Genetic Power Calculator: design of linkage and association genetic mapping studies of complex traits. *Bioinformatics* **19**, 149-150 (2003).

4. Wong, K.C.-Y. *et al.* Long-term metabolic side effects of second-generation antipsychotics in Chinese patients with schizophrenia: A within-subject approach with modelling of dosage effects. *Asian Journal of Psychiatry* **100**, 104172 (2024).
